# Double-blind placebo-controlled food challenges in children with alleged cow’s milk allergy: prevention of unnecessary elimination diets and determination of eliciting doses

**DOI:** 10.1186/1475-2891-12-22

**Published:** 2013-02-08

**Authors:** Wendy M Dambacher, Ellen HM de Kort, W Marty Blom, Geert F Houben, Esther de Vries

**Affiliations:** 1 Department Pediatrics, Jeroen Bosch Hospital, P.O. Box 90153, 5200 ME, `s-Hertogenbosch, The Netherlands; 2 TNO, Zeist, The Netherlands

**Keywords:** Cow’s milk allergy, Cow’s milk protein, Double-blind placebo-controlled provocation, Milk hypersensitivity, Minimum eliciting dose

## Abstract

**Background:**

Children with cow’s milk allergy (CMA) need a cow’s milk protein (CMP) free diet to prevent allergic reactions. For this, reliable allergy-information on the label of food products is essential to avoid products containing the allergen. On the other hand, both overzealous labeling and misdiagnosis that result in unnecessary elimination diets, can lead to potentially hazardous health situations. Our objective was to evaluate if excluding CMA by double-blind placebo-controlled food challenge (DBPCFC) prevents unnecessary elimination diets in the long term. Secondly, to determine the minimum eliciting dose (MED) for an acute allergic reaction to CMP in DBPCFC positive children.

**Methods:**

All children with suspected CMA under our care (Oct’05 - Jun’09) were prospectively enrolled in a DBPCFC. Placebo and verum feedings were administered on two randomly assigned separate days. The MED was determined by noting the ‘lowest observed adverse effect level’ (LOAEL) in DBPCFC-positive children. Based on the outcomes of the DBPCFC a dietary advice was given. Parents were contacted by phone several months later about the diet of their child.

**Results:**

116 children were available for analysis. In 76 children CMA was rejected. In 60 of them CMP was successfully reintroduced, in 2 the parents refused introduction, in another 3 the parents stopped reintroduction. In 9 children CMA symptoms reappeared. In 40 children CMA was confirmed. Infants aged ≤ 12 months in our study group have a higher cumulative distribution of MED than older children.

**Conclusions:**

Excluding CMA by DBPCFC successfully stopped unnecessary elimination diets in the long term in most children. The MEDs form potential useful information for offering dietary advice to patients and their caretakers.

## Background

The symptoms and signs of cow’s milk protein allergy (CMA) are diverse, non-specific and also characteristic of many other childhood diseases. Therefore, it is difficult to correctly identify an adverse reaction to cow’s milk protein (CMP) [1-4]. Children with CMA need a CMP-free diet to prevent allergic reactions. For this, reliable allergy-information on the label of food products is essential to avoid products containing the allergen. On the other hand, both overzealous labeling and misdiagnosis that result in unnecessary elimination diets, can lead to potentially hazardous health situations [2,5-7].

The real prevalence of CMA is only approximately 2-3% in young children, although between 5 and 15% of infants show symptoms suggestive of CMA [8-11]. Allergic reactions to CMP can be IgE-mediated with an acute (within 45 minutes) appearance of symptoms, or non-IgE-mediated, with an intermediate (within several hours) or late (after 24–72 hours) appearance of symptoms [5,12]. Parentally perceived adverse reactions to milk are very common and are the cause of milk-free diets in a substantial number of patients [2,4,7,13-16]. They can cause nutritional inadequacy, growth retardation, eating disorders and psychosocial problems [4,6,7,17]. Excluding CMA in those children who do not have it using a double-blind placebo-controlled food challenge (DBPCFC), the golden standard for the diagnosis of CMA, is important to prevent adverse effects associated with an unnecessary elimination diet. The DBPCFC outcome should also take into account delayed reactions to food. Ignoring these could lead to unjust rejection of the diagnosis of CMA by the physician or persisting conviction in the parents that these late reactions are a reaction to CMP.

In children who do have DBPCFC-proven CMA, a CMP-free diet should be prescribed, irrespective of the underlying immunological mechanism. Reliable allergy-information on the label of food products supports parents to realize this, especially for processed food products because these may (intentionally or unintentionally) contain CMP. To prevent unnecessary labeling of products that contain only trace amounts of CMP to which CMA-patients do not react, knowledge of the minimum eliciting dose (MED) of CMP is required. The MED is defined as the minimal amount of allergen at which an allergic reaction occurs. The MED can be used to quantify the effects of specific amounts of allergens in products, leading to accurate risk assessment and clinically relevant product labeling. This is especially important for children with an acute IgE mediated allergic reaction to cow’s milk, since these reactions are potentially life-threatening. MED data in the youngest age group are hardly available in the literature, whereas these form the largest number of CMA patients.

The primary goal of our study was to evaluate if excluding CMA by a DBPCFC that includes assessment of late reactions prevents unnecessary elimination diets in the long term. Our secondary goal was to determine the MEDs for CMP in children with an acute reaction to CMP (within 2 hours after ingestion), to aid in clinically relevant labeling of food products.

### Patients and methods

Between October 2005 and June 2009, all children with suspected CMA under our regional hospital care who had been using a cow’s milk free diet for at least 4 weeks were prospectively enrolled in a DBPCFC if informed consent was given. Concomitant diseases e.g. atopic dermatitis were no exclusion criterion. Also, no selection was made based on the severity of CMA. At enrollment, symptoms and signs that had led to the suspicion of CMA were obtained from the parents and medical files. Because it is important to prevent unnecessary and to prescribe necessary elimination diets irrespective of the underlying immunological mechanism, and because a positive specific IgE does not prove CMA, the Dutch national protocol for the diagnosis of CMA does not advise specific IgE or skin prick test in the diagnostic evaluation of suspected CMA. Therefore, these were not performed. This study was approved by the medical ethical committee of the Jeroen Bosch Hospital.

The DBPCFC protocol (Table 1) was performed in our pediatric day care ward on two separate days with at least 1 week in between using placebo feeding A and verum feeding B on randomly assigned days. For placebo feeding A the child’s own commercially available hydrolyzed infant formula was used with which the child was symptom free. This formula consisted of either Nutramigen (Mead Johnson, Woerden, the Netherlands), Nutrilon Pepti (Nutricia, Zoetermeer, the Netherlands) or Neocate (Nutricia, Zoetermeer, the Netherlands). Feeding B contained a quantity of cow's milk protein equal to regular infant feedings (1.8 gram per 100 ml); it consisted of the hydrolyzed infant formula used for feeding A and Protifar (Nutricia, Zoetermeer, the Netherlands) in a proportion of 11:3 [18]. Placebo and verum feedings were equal in taste, color and smell. The feedings were packed in identical blinded bottles. The randomization code was packed in sealed, non-transparent envelopes that remained closed until at least one week after the second test day when the reactions had been assessed. The parents, nursing staff, doctors and investigators were unaware of the administered formula’s nature. Acute reactions (within 2 hours after the test) were immediately checked by the physician. Parents noted reactions (within 72 hours after the test) in a home diary. At home, patients continued their CMP free diet until their visit to the outpatient clinic at least one week after the second test day. There, the test was interpreted according to the DBPCFC protocol and a dietary advice was given (Table 1). Parents were contacted by phone several months later about the symptoms and diet of their child.

**Table 1:** The DBPCFC study algorithm

Part 1	The test (performed on two separate days with a 1 week interval)				
	-	No feedings from midnight onwards			
	-	Admittance to pediatric day care ward at 8 AM.			
	-	Physical examination by physician			
	-	DBPCFC schedule			
		Step	Time (in minutes)	Amount (in ml)	Amount (in mg CMP)
		1	0	1	18
		2	20	10	180
		3	40	20	360
		4	60	30	540
		5	80	40	720
		6	100	60	1080
		7	120	90	1620
	-	Physical examination by physician in case of suspected reaction; if confirmed the test is stopped			
	-	Physical examination by physician 20 minutes after last dose			
	-	Clinical observation continued until 1 hour after last dose			
	-	Parents are instructed about home symptoms’ diary			
Part 2	Interpretation of test results				
	-	Visit at outpatient department at least one week after completing DBPCFC with assessment of reactions			
	-	Envelope with randomization code is opened			
	-	Diagnosis CMA is confirmed if symptoms appeared during or within 72 hours after verum feeding and not during or within 72 hours after placebo feeding. These symptoms have to be either identical to the presenting symptoms or severe objective symptoms.			
Part 3	Dietary advice				
	-	CMA: continue a diet free of CMP and repeat challenge in future			
	-	No CMA: reintroduction of CMP over a 4 week period			
		Week	Amount of CMP in feeding		
		1	¼ cow’s milk containing feeding and ¾ hydrolyzed formula		
		2	1/3 cow’s milk containing feeding and 2/3 hydrolyzed formula		
		3	2/3 cows milk containing feeding and 1/3 hydrolyzed formula		
		4	Exclusively cow’s milk containing feeding		
Part 4	Long term follow up				
	-	Interview by telephone about the child’s diet and symptoms			

CMA, cow’s milk allergy; CMP, cow’s milk protein; DBPCFC, double-blinded placebo- controlled food challenge.

Statistical analysis was performed using SPSS 19.0. Two-sided chi-square tests with continuity correction were performed for every single presenting symptom. P-values <0,05 were considered significant.

For determination of the MED, only children with a positive DBPCFC with symptoms within 2 hours after ingestion of CMP were analyzed. The highest dose of CMP at which no symptoms occur (‘no observed adverse effect level’; NOAEL) and the dose of the first reaction (‘lowest observed adverse effect level’; LOAEL) were determined. Individual NOAELs and LOAELs were used to fit a cumulative distribution using the LIFEREG procedure of the SAS system (version 9.1) and applying interval censoring of the data (explained in Taylor et al., 2009) [19-21]. Interval censoring is used when the exact dose that provokes a reaction in an individual is not known, but is known to fall into a particular interval. Using a cumulative distribution makes direct comparison of the distribution of MEDs in populations of variable size possible. The result is a cumulative distribution of the MEDs in which the probability (between 0 and 100%) of the allergic response is presented that occurs at a certain dose (amount of protein intake) less than or equal to this certain dose. For comparison, data from the literature [5,22-24] were fitted using the same method. In determining the individual MED, we used the discrete dose at which the symptoms occur instead of the cumulative dose for several reasons. The interval time between the steps of the protocol is 20 minutes. However, when there is a subjective or mild objective symptom, the next dose is awaited until the symptoms have disappeared. Thereafter, the previous dose is repeated before the next dose is given. This makes the contribution of previous doses to the development of symptoms unclear. Secondly, in a risk assessment a worst case scenario is preferred. Therefore, we assume the discrete dosage is the eliciting dose for the individual patient.

## Results

### Patients

One-hundred-and-twenty-four patients were eligible, of which 51 (41%) were girls. Age at DBPCFC varied from 2.5 months to 134 months (median 7.5 months). In 6 children the DBPCFC was not completed. In 5 of these children, this was because they refused to drink the test feedings. In one child the DBPCFC failed because of an intercurrent infection. One child didn’t meet the inclusion criterion of a CMP free diet for at least 4 weeks. Therefore 116 children are available for further analysis.

### Presenting symptoms

The presenting symptoms of the 124 eligible children are listed in Table 2. Feeding problems were significantly more often present in the DBPCFC-negative group (p=0.046). Swelling was significantly more often present in the DBPCFC-positive group (p=0.023), all four tested patients with this presenting symptom had a positive DBPCFC. The remaining presenting symptoms were not significantly more often present in the DBPCFC-positive or in the DBPCFC-negative children. The number of presenting symptoms was also not significantly different between the two groups. None of the patients presented with an anaphylactic reaction.

**Table 2:** Presenting symptoms of all 124 eligible children

**Symptom**		**Number of patients (%)**
***Gastrointestinal tract***		
	Vomiting	30 (24.2%)
	Diarrhea	17 (13.7%)
	Constipation	14 (11.3%)
	Colic	24 (19.4%)
	Bloody stool	17 (13.7%)
	Abdominal pain	3 (2.4%)
	Feeding problems	15 (12.1%)^†^
***Respiratory tract***		
	Dyspnea and wheezing	13 (10.5%)
***Skin manifestations***		
	Eczema	50 (40.3%)
	Swelling	5 (4.0%)^‡^
	Urticaria	3 (2.4%)
	Erythematous exanthema	10 (8.1%)
***Other***		
	Excessive crying	59 (47.6%)
	Positive family history	8 (6.5%)*

^†^ Feeding problems were significantly more often present in the DBPCFC-negative group (p=0.046).

‡ Swelling was significantly more often present in the DBPCFC-positive group (p=0.023).

*In these 8 children the positive family history was one of the reasons for performing the double-blind placebo-controlled food challenge. In total 65 children (52.4%) had a positive family history.

### Results of DBPCFC

Test results were interpreted according to the type and timing of reactions (Table 3). In 76 children (66%) the diagnosis of CMA was rejected (n=56 <1 year, n=20 1–3 years of age). In 38 (33%) the diagnosis was confirmed (n= 27 <1 year, n= 7 1–3 years, n=4 >3 years of age). Details about the MED and the type of reactions at this dose are listed in Table 4. In 12 of these 38 children (32%) an acute reaction occurred. In 15 of the 38 children (39%) there was a late reaction, and 11 (29%) showed both an acute and a late reaction. In two children an objective acute reaction to placebo feeding alone was observed. The first of these two children (nr 74) developed an acute itching rash on the arms and face after the first dose (18 mg), the second child (nr 118) developed itching eyes and swelling of the mouth after the second dose (180 mg). Although reactions to placebo have been described in the literature, these children were interpreted as having CMA with accidentally exchanged placebo and verum feedings by their treating physician and parents were advised to continue the CMP free diet. They were excluded from further analysis of the MED. In 17 of the 76 children (22%) with a negative DBPCFC a reaction occurred on placebo feeding alone (an acute reaction in 1 child (vomiting), a late reaction in 14 and an acute plus late reaction in 2).

**Table 3:** The 116 successful tests

**Reaction**	**Children**		**Conclusion**	**Reintroduction**
	**Number**	**Percentage**		
No reaction to both feedings	55	47	No CMA	A: 49
				B: 2
				C: 2
				D: 1
				E: 1
Comparable reaction after both feedings	4	3	No CMA	A: 2
				C: 1
				D: 1
Acute reaction after placebo feeding (vomiting)	1	1	No CMA	A: 1
Late reaction after placebo feeding	14	12	No CMA	A: 8
				D: 5
				E: 1
Acute and late reaction after placebo feeding	2	2	No CMA	D: 2
Objective acute reaction after placebo feeding alone	2	2	CMA; interpreted as accidentally exchanged placebo and verum feedings by treating physician	
Acute reaction after verum feeding	12	10	CMA	
Late reaction after verum feeding	15	13	CMA	
Acute and late reaction after verum feeding	11	10	CMA	

CMA, cow’s milk allergy; A, successful reintroduction; B, parents refused to reintroduce cow’s milk; C, reintroduction stopped without medical advice; D, reintroduction stopped with medical advice; E: no information available on reintroduction.

**Table 4:** LOAEL symptoms of the DBPCFC-positive children

**Patient**	**Age (months)**	**Sex**	**Amount of CMP (mg)**	**Symptoms**
***Acute reactions***				
29	7,5	F	?	ery
30	134	M	?	os, nau
35	115	M	18	sw
63	4	M	18	ery
108	42,5	F	18	os, vom, sw
34	10	M	180	ery
90	26	F	360	ery, ur
42	14,5	M	540	ur
93	3	M	540	col
6	3,5	M	720	ery
107	7	M	720	cry, vom
14	5,5	M	1080	ery, ur
85	5	M	1080	dia
104	119	M	1080	ap, nau
47	11	M	1620	ery, vom, dysp, whe
54	8,5	F	1620	vom
59	7,5	M	1620	ery
66	24	F	1620	ery
87	7	F	1620	ec
95	6	M	1620	cry, dia
114	11	F	1620	dia, ery, cry, agi
121	17	M	1620	ery, ur
123	4	F	1620	agi, fp
***Late reactions***				
9	7,5	F	1620	dia, cry
19	12,5	M	1620	sp, ery
21	6	M	1620	ec, con
27	5,5	M	1620	dysp
32	5,5	M	1620	ec, cry
37	2,5	F	1620	agi
39	5	F	1620	cry
62	13	F	1620	col, cry, con
65	7	F	1620	con
73	7	M	1620	agi, cry, dia
76	15,5	F	1620	cry, sp
79	7,5	F	1620	vom, cry
86	5	F	1620	dia
103	4	M	1620	agi
111	4,5	F	1620	con

DBPCFC, double-blinded placebo-controlled food challenge; LOAEL, lowest observed adverse effect level; agi, agitation; ap, abdominal pain; col, colic; con, constipation; cry, crying; dia, diarrhea; dysp, dyspnea; ec, worsening eczema; ery, erythema; fp, feeding problems; itch, itching; nau, nausea; os, oropharyngeal symptoms; sw, swelling; ur, urticaria; vom, vomiting; whe, wheezing; sp, sleeping problems. Amounts of 18, 180, 360, 540, 720, 1080 and 1620 mg CMP correspond with 1, 10, 20, 30, 40, 60 and 90 ml test feeding respectively.

### Reintroduction of cow’s milk

Follow-up was performed by contacting parents by telephone several months after the reintroduction. When we were unable to reach parents by telephone, information was gathered from the medical file. Information on reintroduction was available in 74 of the 76 DBPCFC negative children (Table 3) with a mean follow-up of 12 months (range 1–31 months). In 60 of the 74 children (81%) in whom the diagnosis of CMA was rejected, cow’s milk was successfully reintroduced permanently. In 9 children (12%) symptoms reappeared upon reintroduction of cow’s milk. Symptoms included crying, worsening of eczema and diarrhea. The symptoms disappeared with re-elimination of cow’s milk from the diet. Therefore, they were interpreted by the treating physician as an allergic reaction to cow’s milk, with a false negative DBPCFC, probably due to reactions that only become apparent upon prolonged feeding with high doses of CMP. In 7 of these 9 children a reaction to placebo feeding alone had been observed that was equal to the presenting symptoms. These reactions consisted of colic, diarrhea, vomiting, eczema, erythema, itching, crying and sleeping problems. In one there was a comparable reaction to both feedings (diarrhea, colic), the other child had no reaction to either of the feedings. In 5 of the 74 children (7%) parents refused or stopped reintroduction.

### MED

**Figure 1:**
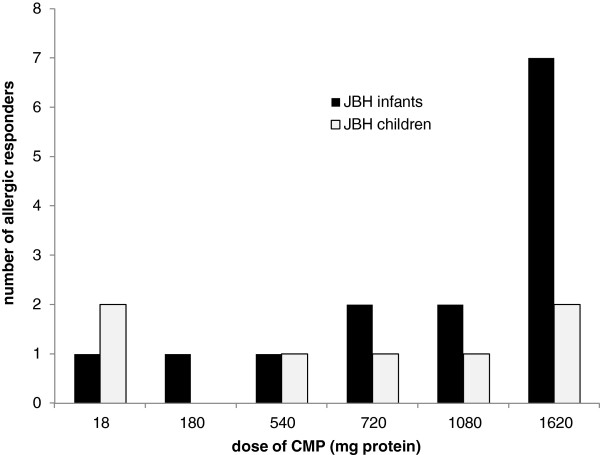
**Responders with an acute reaction at each dose.** The number of cow’s milk allergic children with an acute reaction of the Jeroen Bosch Hospital at each dose of cow’s milk (expressed as CMP). Amounts of 18, 180, 360, 540, 720, 1080 and 1620 mg CMP correspond with 1, 10, 20, 30, 40, 60 and 90 ml test feeding respectively. JBH_children is the patient selection of DBPCFC-positive children older than 12 months. JBH_infants consists of DBPCFC-positive children aged 0 – 12 months. See Table 4 for details on each individual.

**Figure 2:**
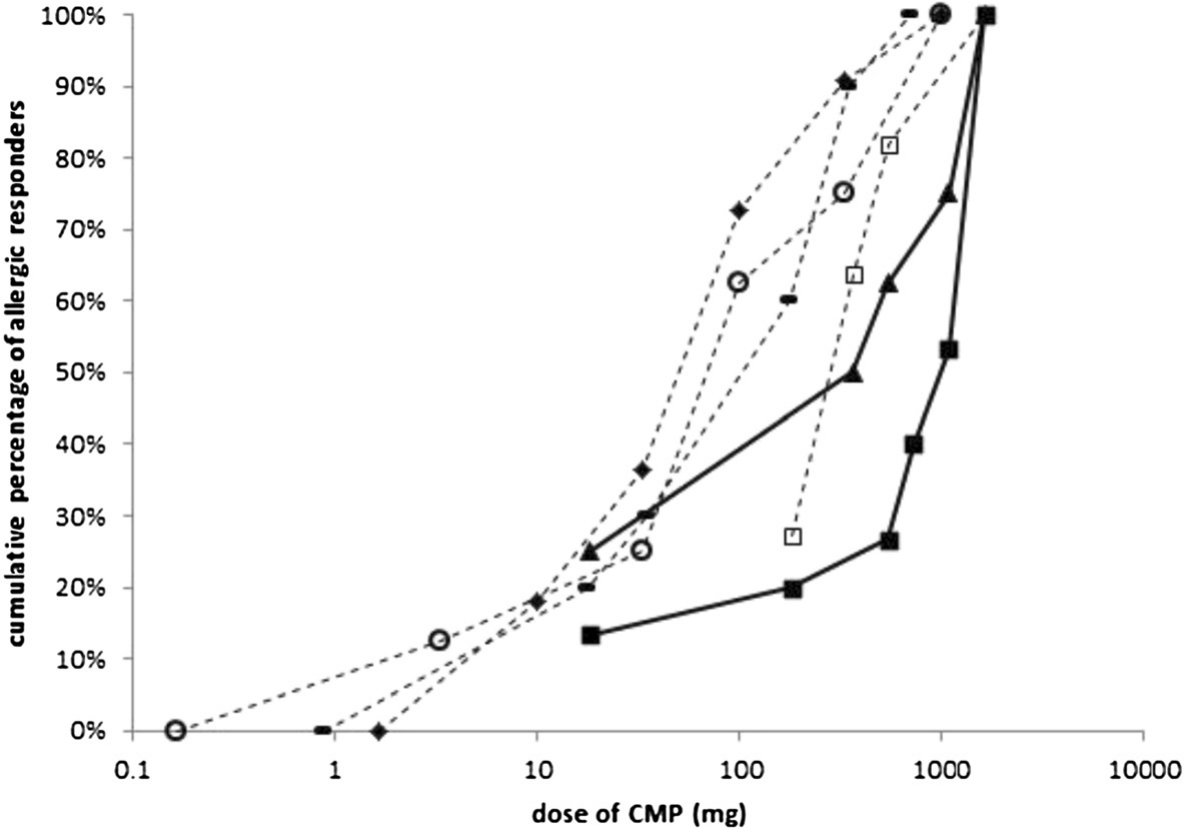
**Cumulative MEDs for an acute allergic reaction to cow's milk protein in the JBH population compared to various CMA studies.** Cumulative probability distribution based on individual MEDs for cow’s milk (expressed as CMP) for cow’s milk allergic children of the Jeroen Bosch Hospital and for populations from the literature [5,22-24]. 100 mg CMP corresponds with 5,6 ml of our test feeding. Two distributions were compiled for the JBH population. JBH_children is the patient selection of DBPCFC-positive children older than 12 months. JBH_infants consists of DBPCFC-positive children aged 0 – 12 months. See also Table 5. CMA, cow’s milk allergy; CMP, cow’s milk protein; JBH, Jeroen Bosch Hospital; MED, minimum eliciting dose. ▲ JBH_children; ■ JBH_infants; □ Flinterman et al. (2006); ▬ Baehler et al. (1996); ♦ Caminiti et al. (2009); ○ Patriarca et al. (2002).

23 children showed a positive DBPCFC with an acute reaction within 2 hours after ingestion of CMP. This excludes the two children with an acute reaction to placebo feeding who were interpreted as having CMA by their treating physician. Information on NOAEL and LOAEL was unavailable for 2 children due to incomplete medical files. Therefore, 21 children were available for determining the MEDs. Figure 1 shows the number of allergic responders at each MED in this population of 21 children (the Jeroen Bosch Hospital population; JBH population). For comparison between age groups, the total population was divided in two subgroups according to age (0–12 months, n=14; >12 months, n=7). To enable comparison of the two populations the individual MED are presented as a cumulative probability distribution (Figure 2). Data from the literature derived from the study populations of Flinterman et al. (n=11) [5], Baehler et al. (n=16) [22], Caminiti et al. (n=13) [23] and Patriarca et al. (n=8) [24] are represented in the same figure. Detailed information on the populations represented in Figure 2 is shown in Table 5. Figure 2 shows that the highest cumulative distribution of MEDs was in our study subgroup of children aged 0 – 12 months. The cumulative MED distribution of our population of children aged > 12 months group is comparable to the patient groups described in the literature.

**Table 5:** **Information on the populations represented in Figure**1

**Study population**	**Hospital**	**Patient selection**	**Number of patients**	**Mean age in years (range)**
JBH_infants	Regional hospital	All children with suspected CMA aged 0 – 12 months.	14	0,6 (0,3 – 0,9)
JBH_children	Regional hospital	All children with suspected CMA aged > 12 months	7	4,2 (1,2 – 9,9)
Flinterman et al.[5]	Tertiary referral centre	Children sensitized to CMP and with AEDS as reason for prolonged CM elimination.	11	4,1 (1,8 – 10,3)
Baehler et al.[22]	Tertiary referral centre	Children with suspected CMA. All children with chronic atopic dermatitis are excluded.	16	3,1 (0,7 – 8,8)
Caminiti et al.[23]	Tertiary referral centre	Children with severe IgE-mediated CMA	13	8 (5 – 10)
Patriarca et al.[24]	Tertiary referral centre	Children with CMA from the outpatient clinic offered an oral desensitization.	8	9 (5 – 15)

AEDS, atopic eczema dermatitis syndrome; CM, cow’s milk; CMA, cow’s milk allergy; CMP, cow’s milk protein; JBH, Jeroen Bosch Hospital.

## Discussion

An accurate diagnosis of CMA is important to reduce the number of children on inappropriate diets. Many studies using DBPCFC with CMP to evaluate the incidence of CMA have been retrospective [3], and/or did not include late reactions [25]. None included long-term follow up to assess if parents continued to follow the medical advice based on the DBPCFC. In our study DBPCFC led to the long-term use of an appropriate diet based on the presence or absence of CMA in 100 (88%) of 114 children tested (intention-to-treat analysis; information on long term diet was unavailable in 2 children).

Besides acute reactions, we also studied late reactions which typically develop within 24 to 72 hours after ingesting CMP. When late reactions are described in the literature, they form a substantial part of the positive test results, ranging from 20% to 60% [22,26]. When only acute reactions are included, as in the study by Schade et al. [25], a much lower incidence of CMA is found than mentioned in the literature. In our study population, 37.5% of the 40 children with CMA developed a late reaction alone. A limitation of our study is, that the only information we have about late reactions comes from the parents; it is hardly feasible to hospitalise children for a total of 6 days to perform a DBPCFC. However, only symptoms that were identical to the original presenting symptoms that occurred after verum feeding but not after placebo feeding were interpreted as a positive DBPCFC. Systematically ignoring late reactions in DBPCFC would lead to an unjust rejection of the diagnosis of non-IgE-mediated CMA.

The presenting symptom of swelling was significantly more often present in the DBPCFC positive group. None of these children had a negative test. This is not surprising, since swelling is usually an immediate IgE-mediated hypersensitivity reaction. In these cases, the diagnosis is more easily made on clinical presentation. However, presenting symptoms of urticaria, erythema, vomiting and respiratory tract symptoms, which can also be interpreted as IgE-mediated reactions when occurring as an immediate reaction, were not significantly different between the two groups. This emphasizes the need for a DBPCFC for diagnosing or excluding CMA.

Reactions to placebo are described in the literature. Vlieg-Boerstra et al. found them in 12.9% of all their DBPCFC tests and in 5/43 (11,6%) of their cow’s milk DBPCFC’s [27]. Hospers et al. describe a reaction to placebo feeding in 24% of their tests [3]. We also found them in 17 children (22%). The precise cause for this is not known. We cannot fully exclude the possibility that placebo and verum feedings were accidentally exchanged in some of the cases with a reaction to placebo feeding alone, because 7 DBPCFC negative children in our study group who had developed a reaction after placebo feeding alone were later interpreted as having CMA based on recurrence of symptoms after the reintroduction of CMP. However, a reaction to placebo feeding alone occurred in 9 additional DBPCFC negative children in whom CMP was successfully reintroduced.

A possible explanation for a false negative result is the possibility that the threshold to respond is higher than the dose achieved during the challenge, i.e. larger quantities of allergen are needed to produce a reaction. Sicherer et al. [28] studied the quantity of food that elicited a reaction during DBPCFC in children with atopic dermatitis. Of 117 children (median age 5 years 9 months) with positive reactions to CMP, in 12% the reaction occurred after the final test dose of 2 to 2,5 grams or during open challenge. These children received a total of 8 to 10 grams of CMP. In our study the final dose consisted of 1,6 grams and a total of 4,5 grams of CMP was ingested during the test. So, it could be that some children in our study did not receive a high enough dose to produce a reaction.

Unfortunately, the data represented by Sicherer et al. is not detailed enough for us to determine a cumulative distribution of the MEDs of their study group for comparison with our study group. Recent research has explored the importance of having adequate MED-data available for population risk assessment purposes which makes optimal use of all available information, including the dose distribution of MEDs within the allergic population [29,30]. However studies that are developed to determine MEDs often only describe the lowest MED within the population encountered, whereas a distribution of MEDs within that population is not established [31]. Flinterman et al. [5], Baehler et al. [22], Caminiti et al. [23] and Patriarca et al. [24] however, do represent data in their studies describing allergic reactions to CMP which can be used for determining the cumulative distribution of MEDs in their population.

It is striking that our subgroup of infants aged ≤ 12 months has a higher cumulative MED distribution than the children aged > 12 months. This suggests that infants have a higher MED than older children, and therefore will react only to higher amounts of CMP. This is supported by the studies from the literature used for comparison. The cumulative MED distributions based on these studies are also lower than the cumulative MED distribution of our infant group. As can be seen in Table 5, the age distribution of the children described in the literature is comparable to our subgroup of children aged > 12 months. However, there are some important differences between our study and the studies in the literature, which makes comparison difficult. At first, we performed our study in a regional hospital. All the studies from the literature were performed in a tertiary referral centre, which can lead to a different patient selection. Patients visiting a tertiary referral centre may have a more severe CMA, and therefore a lower MED. Secondly, our study is the only study that included all children with suspected CMA, without selection based on the presence or absence of atopic dermatitis. Also no selection was made based on the severity of CMA.

The population of Flinterman et al. [5] is most similar to our subgroup of children considering the age distribution. However, they included only children with atopic dermatitis. The children described by Baehler et al. [22] are somewhat younger, however they excluded all children with co-existing atopic dermatitis. The children in the study groups of Caminiti et al. [23] and Patriarca et al. [24] are not only older than our population, but also consist of a selected patient group. These two studies were performed to investigate the effect of oral desensitization, and children with a ‘severe’ CMA were selected. It is not surprising that these children have a lower MED distribution. Therefore, our study seems more representative for the general population of children with CMA compared to the other studies mentioned above. Our study is the only one that included enough infants to allow a separate distribution for children aged ≤ 12 months. With the possible exception of the study by Baehler et al., in which the age distribution is not clearly described, none of the studies included children under the age of 12 months.

A recent paper by Brand et al. [32] states that the individual MED remains fairly constant over time, however we found no other studies in the literature to confirm this statement. They also state that 75% of infants with CMA are cow’s milk tolerant by the age of 1 year. 90% are cow’s milk tolerant by the age of 4 years. A study by Host et al. [33] in 2002 investigated the natural history of CMA. They found a recovery of CMA in 56% of patients at 1 year, 77% at 2 years, 87% at 3 years, 92% at 5 and 10 years and 97% at 15 years of age. In our study group, older children have a lower cumulative distribution of MED than the infants aged 0 – 12 months. An interesting discussion point is whether this means that the infants with a higher MED will become cow’s milk tolerant and infants with a lower MED will remain allergic to cow’s milk. Another hypothesis is that there is some kind of selection bias. Infants in general consume more milk than older children. Therefore both infants with a relatively high MED and a low MED might seek medical attention in contrast to older children who would just start consuming less cow’s milk products and don’t seek medical advice unless they have a lower MED.

Further follow-up studies are needed to confirm these hypotheses and to investigate whether the individual MED remains constant over time. At this point, the cumulative distribution of the MED in a population can only be used for population risk assessment purposes.

## Conclusion

By excluding CMA by DBPCFC most parents are convinced the symptoms of their child are not caused by CMP and are willing to permanently stop an unnecessary elimination diet. This study shows that it is important to include late reactions to CMP in the DBPCFC test. By ignoring these late reactions the diagnosis of CMA would have been rejected unjustly in 37,5% of the children in our study. Also, by ignoring late symptoms parents could remain convinced that these symptoms are attributable to CMP and therefore would unnecessarily continue an elimination diet.

When CMA is proven, the DBPCFC can be used to determine the MED and the cumulative MED distribution. The MEDs form potential useful information for offering dietary advice to patients and their caretakers and for population risk assessment purposes. Our study shows that older children have a lower cumulative MED distribution than the infants in our study group, and thus react to smaller amounts of CMP. Further studies are needed to investigate if the individual MED can also be used to predict the chance of an infant becoming cow’s milk tolerant.

## Abbreviations

CMA: Cow’s milk allergy; CMP: Cow’s milk protein; DBPCFC: Double-blind placebo-controlled food challenge; LOAEL: Lowest observed adverse effect level; MED: Minimum eliciting dose; NOAEL: No observed adverse effect level

## Competing interests

There are no conflicts of interests.

## Authors’ contributions

WD participated in the acquisition, analysis and interpretation of data and drafted the manuscript. EK participated in the design of the study, the acquisition of data, and helped to draft the manuscript. WB and GH participated in the design and analysis of the data concerning the minimum eliciting dose. EV conceived of the study and participated in its design and coordination and helped to draft the manuscript. All authors read and approved the final manuscript.
